# Acclimation responses of macaw palm seedlings to contrasting light environments

**DOI:** 10.1038/s41598-018-33553-1

**Published:** 2018-10-17

**Authors:** Adriel N. Dias, Advanio I. Siqueira-Silva, João P. Souza, Kacilda N. Kuki, Eduardo G. Pereira

**Affiliations:** 1 Programa de Pós-graduação em Manejo e Conservação de Ecossistemas Naturais e Agrários, Universidade Federal de Viçosa (UFV), Campus Florestal, Florestal, Minas Gerais Brazil; 2 Instituto de Ciências Biológicas e da Saúde, UFV, Campus Florestal, Florestal, Minas Gerais Brazil; 3 Departamento de Fitotecnia, UFV, Campus Viçosa, Viçosa, Minas Gerais Brazil; 4 Present Address: Universidade Federal do Oeste do Pará (UFOPA), Campus Universitário de Juruti, Juruti, Pará Brazil

## Abstract

The photosynthetic adjustments of macaw palm (*Acrocomia aculeata*) were evaluated in 30-day-old seedlings exposed to high and low light environments, and sudden transference from low to high light and comparisons were made with the hardening protocol used in nurseries. Furthermore, we evaluated the responses to long-term exposure (265 days) to high and low light environments. Macaw palm seedlings exhibited an efficient mechanism that maximized light capture under scarce conditions, and dissipated excess energy to avoid damaging to the photosystem II under high light. The seedlings showed low saturation irradiance but no photoinhibition when exposed to excess light. When grown under low light intensities, seedlings presented higher photochemical efficiency and minimized the respiratory costs with positive carbon balance at lower irradiance than hardened seedlings did. The hardening procedure did not appear to be an advantageous method during seedling production. Long-term exposure to either low or high light did not cause significant leaf anatomical adjustments. However, the low light seedlings showed higher leaf area and chlorophyll content than those exposed to higher light intensity did, which enabled shaded seedlings to maximize the captured light. Furthermore, the high non-photochemical dissipation allowed rapid acclimation to excessive light exposure. These responses allow macaw palm cultivation and establishment in very different light environments.

## Introduction

Light is an essential resource for the growth and development of plants, and the main determining factors are intensity, quality, duration, and distribution of the photoperiod. However, light is also a limiting resource to the growth and reproduction of some palm species^1^. To cope with shaded environments, plant species generally have two adaptive strategies, namely shade avoidance and tolerance^2^. Those that avoid shade (shade intolerant, light demanding) use the resources available in the environment to grow toward the light, as opposed to shade-tolerant species that preferentially exhibit efficient absorption and use of the scanty light energy under shade conditions. Acclimation to environments with low light intensities may involve changes in the chloroplast, leaf and plant levels^3^. Generally, neotropical palm species are shade tolerant, and they show some important leaf responses such as low compensation irradiance, epidermal chloroplasts, and horizontally oriented leaves with a long lifespan^4^.

The leaf, the main vegetative plant organ for capturing light, is considered highly plastic and, consequently, it is most evaluated in studies of the effects of abiotic stresses on plants^5^. The ability of plants to acclimate to different irradiances is a function of the morphophysiological adjustments of their leaves. These responses may vary among plant species or even with different development stages of a single species^6^. The amount of light that reaches leaves during development can cause structural adjustments in these organs, with significant changes in the thicknesses of the mesophyll and leaf blade, stomatal density, and the volume of the intercellular spaces^7,8^. There may also be changes in the area and mass of leaves^9,10^, and the efficiency of light interception depending on leaf orientation, shape, and size^11^.

In addition to the morphoanatomical plasticity, some plant species can adjust their photosynthetic responses to changes in irradiance, and these physiological variables play a decisive role in the acclimation of the plant to a shaded or full-sunlit environment^6,7,9^. The ability of plants to allocate biomass and survive under the most diverse lighting conditions depends on their photosynthetic capacity^11^, preventing photoinhibition under exposure to excessive light, or maximizing light capture when this resource is scarce^12^. A sudden increase in irradiation in the understory due to the opening of clearings in the forest canopy can result in physiological stress and determine the distribution pattern of some palm species^9^. A similar seasonal effect can also be observed in semi-deciduous tropical forests when the leaves fall from the canopy, which increases the light intensity that reaches the lower stratum^13^. This increased light intensity is a disadvantage to shade-tolerant species, whereas it favors the pioneers or the light-demanding species^13^. Although palms are a regular fixture of the tropical vegetation, most information about their carbon physiology is limited to crop species including *Eleais guineensis* and *Cocus nucifera*.

The oleaginous palm *Acrocomia aculeata* (Jacq.) Lood. Ex Mart. (Arecaceae), also known as macaw palm, is found in savanna-like vegetation, semi-deciduous seasonal forests, and deforested areas, among other physiognomies of Central and South America^14,15^. It preferentially occurs in areas of higher natural soil fertility but also can occur in sandy and low-fertile soils^16^. Because of its high productivity and oil traits, the palm has great potential for the industrial sectors in the production of vegetable oil, for pharmaceutical, cosmetics, food, and biofuel development^17^. Native to the neotropics, the macaw palm is classified as a pioneer species^18^, and it has remarkably drought tolerance^19^. Despite being described as a heliophytic species, little is known about the acclimatory responses of young macaw palm plants to light.

The standard protocol to overcome dormancy and promote germination of macaw palm seeds^20^ was established by Motoike, *et al*.^21^ (Patent registration: PI0703180-7 A2). The recommended initial seedling cultivation process consists of using pre-germinated seeds in two consecutive stages: the pre-nursery stage, which extends from pre-germination to the appearance of the first pair of lanceolate (split) leaves and the nursery stage that extends to the appearance of the second pair of pinnate (mature) leaves. Starting from this point when the seedlings are approximately 1 year old, field planting can begin^22^. Generally, after the formation of the first eophyll in the pre-nursery stage, a hardening procedure is empirically used to promote the robustness of seedlings and enhance their capacity to acclimate to excessive light, thereby preventing photoinhibitory stress in the nursery and hastening development in the field^23^. During the hardening procedure in macaw palm, the young seedlings are daily transferred from the shading cloth structure of the pre-nursery (low light environment) to a higher light intensity, expect in the hours when irradiance levels are at the highest, i.e., between 10:00 am–4:00 pm. However, currently, no studies have evaluated the morphological and physiological responses of young macaw palm plants to contrasting light environments.

Field-grown macaw palm plants exhibit high photosynthetic efficiency under high irradiances and can maintain carbon assimilation by low compensation and saturation irradiance^18^. Therefore, we investigated the hypothesis that young *A*. *aculeata* plants have high acclimation capacity and low susceptibility to photoinhibition when exposed to contrasting irradiance conditions. The objective of this study was to evaluate the photosynthetic and morphoanatomical adjustments of the macaw palm seedlings to different light intensities.

## Methods

### Plant material and experimental conditions

The study was conducted at the Federal University of Viçosa, *Campus* Florestal (19°52′S, 44°25′W). The seeds of *A*. *aculeata* were collected in the central region of the State of Minas Gerais, Brazil. The protocol for overcoming dormancy and the germination process was performed according to the method of Motoike, *et al*.^21^. The seedlings were transferred to tubes (180 cm^3^) containing a commercial substrate Tropstrato® HT containing 1 kg m^−3^ of single superphosphate fertilizer. During the first 30 days, the seedlings were grown under low light environment (maximum irradiance of 320 µmol m^−2^ s^−1^) until the emergence of the first eophyll. Subsequently, two assays with different durations of the treatment exposure were conducted.

In the first assay, during the pre-nursery stage, 1-month-old macaw palm seedlings were exposed for 30 days to the following three treatments. (i) The seedlings were grown in a greenhouse under high light conditions throughout the entire period (maximum irradiance of 1030 µmol m^−2^ s^−1^). (ii) The seedlings remained shaded (low light condition) under a 2.10 m high × 1.80 m wide structure, covered with a shade cloth typically used in nurseries (maximum irradiance of 320 µmol m^−2^ s^−1^). (iii) The seedlings were subjected to the hardening procedure and were maintained under low light during the day with the highest irradiance (10:00 am–4:00 pm), and then they were transferred to high light conditions prior to or after this period. In addition, after the first 30 days, some of the seedlings that were under the low light treatment were exposed to a new treatment: (iv) the seedlings were directly transferred to high light condition, without the process of hardening. Immediately after this treatment commenced, physiological and morphological evaluations of seedlings from all treatments were performed on the second split-leaves with typical parallel veins^24^, which developed after the eophyll under each lighting condition.

In the second assay, at the nursery stage, young macaw palm plants (3-month-old) from the high light exposure and low light treatments were transplanted into polyethylene bags (5 L) filled with substrate, containing a soil mixture:sand:cattle manure (2:1:1), enriched with 1 kg m^−3^ dolomitic limestone, 6 kg m^−3^ single superphosphate, 1 kg m^−3^ ammonium sulfate, and 0.30 kg m^−3^ potassium chloride. The trial consisted of long-term exposure (over 265 days) of the seedlings to the two contrasting light treatments in the same setting as the first assay. Throughout the exposure period, the chlorophyll *a* fluorescence, photosynthetic pigment content, specific leaf area (SLA), growth variables, and leaf anatomy were evaluated.

### Evaluation of photosynthetic responses of macaw palm seedlings under contrasting irradiances

In the first assay (short-term exposure to contrasting light conditions), we evaluated the net photosynthetic rate (*A*, µmol m^−2^ s^−1^), stomatal conductance (*g*_s_, mol m^−2^ s^−1^), transpiration rate (*E*, mmol m^−2^ s^−1^), the ratio between internal and external CO_2_ concentration (*Ci*/*Ca*), and instantaneous water use efficiency (*W*_t_ = *A*/*E*, µmol_CO2_ mmol_H2O_ m^−2^ s^−1^) in seedlings in all treatment groups for 9 days, from the day the seedlings were transferred to high light conditions. Gas exchange measurements were performed between 08:00 am and 12:00 pm using an infrared (IR) gas analyzer (model LI-6400XT, Li-Cor Inc., Lincoln, NE, USA) at light intensity of 1,000 µmol photons m^−2^ s^−1^ provided by a light source (6400-40 Leaf Chamber, Li-Cor Inc., Lincoln, NE, USA) for all plants. The CO_2_ concentration was set to 400 μmol mol^−1^ using a CO_2_ injector system (6400-01, Li-Cor Inc., Lincoln, NE, USA). Other climatic parameters were not controlled during the assay (mean ambient temperature of 27 ± 3 °C and mean vapor pressure deficit of 2.2 ± 0.3 kPa).

The chlorophyll *a* fluorescence variables *F*_0_ and *F*_v_/*F*_m_ were measured using the Mini-PAM modulated pulse fluorometer (Heinz Walz, Effeltrich, Germany) in the same leaflet region where the gas exchange was measured in plants in the first experiment. Briefly, the leaflets were acclimatized in the dark for 30 min and then exposed to a weak pulse of red light (0.15 μmol m^−2^ s^−1^) to determine the *F*_0_. Then, a pulse of saturating light at 12000 μmol photons m^−2^ s^−1^ was applied for 0.8 s, to determine the maximum fluorescence (*F*_m_). The obtained data were used to estimate the *F*_v_/*F*_m_ = (*F*_m_ − *F*_0_)/*F*_m_)^25^. The chlorophyll content index was evaluated in the first assay using the portable chlorophyll meter ClorofiLOG (Falker, Porto Alegre, RS, Brazil) before the fluorescence analysis and three measurements were performed on each leaf blade.

In the second assay (long-term exposure to constant high light and low light), foliar pigments were quantified using dimethyl sulfoxide (DMSO) as the extractor. Briefly, at the end of the trial, leaf discs with an average dry mass of 7.5 mg, were removed from each replicate evaluated and incubated in 5 mL saturated DMSO in calcium carbonate (CaCO_3_). To speed up the pigment extraction, the samples were kept in water bath at 65 °C for 36 h, and the absorbance was read at 480, 649.1, and 665.1 nm. The photosynthetic pigments (chlorophyll *a*, chlorophyll *b*, and carotenoids) were determined according to the method of Wellburn^26^. The results were expressed in mg g_DW_^−1^.

### Evaluation of photosynthetic light response curves in macaw palm seedlings

Light response curves of the net *A* and effective quantum yield of photosystem II (ϕ_PSII_) were constructed for seedlings from the first experiment on day 14 after the plants were transferred to full sunlight conditions, using the autoprogram function of the LI-6400XT IR gas analyzer attached to a fluorescence chamber. Briefly, the leaflets were exposed to decreasing actinic light intensities (2000, 1500, 1000, 500, 200, 100, 50, 20, and 0 µmol m^−2^ s^−1^) for a minimum and maximum time of 120 and 200 s, respectively for each light intensity, and a CO_2_ concentration of 400 μmol mol^−1^ under the same conditions described previously. The light response curves of the net *A* obtained were adjusted using the model that best fit the data, the hyperbolic rectangular model^27^, using *Microsoft Excel* 2013^28^. We evaluated the dark respiration (*R*_*D*_), light compensation point (*I*_comp_), maximum net *A* (*A*_max_), the light saturation point beyond which there is no significant change in net photosynthesis (*I*_max_), and the maximum quantum yield (ϕ_,_ taking the linear response of *A* between *I*_comp_ and 200 µmol photons m^−2^ s^−1^). The light response curves of the ϕ_PSII_ were adjusted using exponential decay and linear equations where appropriate.

In the second assay, rapid light response curves were obtained by using the Mini-PAM fluorometer to determine the instantaneous capacity of photosynthetic response to excessive solar radiation in macaw palm seedlings. At first, the leaflets were dark-acclimated for 30 min to determine *F*_0_ and *F*_m_. Then, the leaflets were exposed to the light conditions where plants grown for at least 10 min and the light response curve was started. The leaflets were exposed to increasing actinic light intensities (0–2000 µmol m^−2^ s^−1^) during nine 10-s intervals, and the *steady-state* fluorescence (*F*_s_) and maximum light-adapted fluorescence (${F^{\prime} }_{m}$) were measured. Based on these values, we calculated the ϕ_PSII_^25^ and the quantum yield of regulated (ϕ_NPQ_) and non-regulated (ϕ_NO_) energy dissipation^29^. The photochemical quenching (*q*_L_)^30^ and non-photochemical quenching (*q*_N_)^31^ were also calculated. The values for minimum fluorescence in the light-adapted state (${F{\rm{^{\prime} }}}_{0}$) were obtained according to Oxborough and Baker^32^. The apparent electron transport rate (*ETR*) was calculated as: *ETR* = ϕ_ΙΙ_ × PAR × *I*_A_ × 0.5; where PAR = photosynthetically active radiation, *I*_A_ = the leaflet absorptivity coefficient, and 0.5 = fraction of excitation energy distribution in PSII^33^.

### Evaluation of photoinhibition and contribution of non-photochemical components to de-excitation of PSII in macaw palm seedlings

The extension of photoinhibition and recovery capacity were evaluated in macaw palm seedlings in the first assay on day 20 after the commencement of the direct transfer treatment following the protocol of Zivcak, *et al*.^34^ with modifications. In pre-dawn, the *F*_v_/*F*_m_ measurements were determined using the Mini-PAM fluorometer. Then, the macaw palm leaves were exposed to 50 μmol photons m^−2^ s^−1^ for 5 min, and soon after that, a light pulse was administered to determine the ϕ_PSII_. Then, the leaves were exposed to the photoinhibitory condition with light intensity of 3000 μmol m^−2^ s^−1^, (from an external light source 2050-HB Heinz Walz, Effeltrich, Germany). A filter eliminating wavelengths above 710 nm and a ventilator were used to exclude IR radiation and over-heating of the leaf. Light saturating pulses (12000 μmol photons m^−2^ s^−1^) were applied at each 10-min interval over a 40-min period. Subsequently, the plants were allowed to recover from the photoinhibitory treatment in the dark, with photochemical efficiency measurements at 1, 15, and 30 min. Then, 24 h after the high light treatment, a new *F*_v_/*F*_m_ evaluation was performed at the pre-dawn stage to characterize the extent of photoinhibition.

The contribution to the different components of *q*_N_ to the de-excitation mechanisms was determined using dark relaxation kinetics of fluorescence in plants from the second experiment. First, the *F*_0_ and *F*_m_ were determined at dawn. Then, in the morning (08:00 am) the leaflets were exposed for 2.5 min to 3000 µmol photons m^−2^ s^−1^. After 1, 5, 20, and 90 min exposure to excess irradiance, the *F*_0_ and *F*m were measured^35^. The three components of *q*_N_, energy-dependent quenching (*q*_E_), state transition quenching (*q*_T_), and photoinhibitory quenching (*q*_I_), have different relaxation times. The fastest, relaxation time (2–5 min.) indicates the dissipation through *q*_E_, the intermediate relaxation time (12–20 min), indicates the dissipation by *q*_T_; and the slow relaxation time (>40 min) indicates the degree of dissipation by the photoinhibition mechanism^35^.

### Morphoanatomy traits and leaf micromorphometry of macaw palm seedlings grown under contrasting light conditions

In the first assay, the leaf area was determined on day 27 after the direct transfer treatment started and periodically throughout the long-term shading in the second experiment, using a portable leaf area meter (LI 3000C, Li-Cor Inc., USA). In the second assay, the diameter and length of the stipe were also evaluated using an analog caliper and millimeter ruler, respectively.

To evaluate the SLA, a leaflet was collected from each plant in the second assay. The leaf area (LA) was determined as shown previously. The leaflets were then dried at 75 °C until a constant mass was obtained (48 h) for the dry mass (DM) quantification. The SLA was calculated as the ratio of the LA to DM.

For the light microscopy structural analyses, samples from the middle region of fully expanded leaflets grown under long-term low and high light conditions (second experiment) were fixed in formaldehyde-acetic-alcohol (FAA) 70 solution (37% formaldehyde, 100% glacial acetic acid, and 70% ethanol, 1:1:18, v/v/v)^36^ for 48 h. They were then dehydrated in an ethanol series and embedded in Leica historesin (Leica Microsystems Inc., Heidelberg, Germany), according to the manufacturer’s recommendations. Cross sections (5 μm) were cut using a rotary microtome, stained with 0.05% toluidine blue in 0.1 M phosphate buffer pH 6.8^37^, and mounted in Entellan®. The images were acquired using a photomicroscope (model BX41 TF, Olympus Optical, Tokyo, Japan) equipped with an image capture camera (model SC 30, Olympus Soft Imaging Solutions GmBH, Munster, Germany).

For the micromorphometric analyses, three leaflets were collected from each plant (n = 5), fixed in FAA 70 solution, and one sample from each replicate was randomly processed, following the methodology of the structural characterization. The height of the epidermal cells, the hypodermis of both faces of the leaflet, and the thickness of the mesophyll and the limbus were measured. The six parameters were measured three times per histological section, in five photomicrographs of each replicate (n = 5) of the shading and full sunlight conditions using the AxioVision 4.9.1 software, Carl Zeiss Microlmaging GmbH, Jena, Deutschland.

### Statistical analysis

A randomized block design (n = 5) was used for all the experiments, and each experimental unit was composed of a macaw palm seedling. The data were analyzed using an analysis of variance (ANOVA), where the means were previously evaluated using the Breusch-Pagan homogeneity of variance test and the Shapiro-Wilk variance homogeneity test (5% probability) using the statistical program R version R i386 3.1.2. Comparisons between means were performed using the Tukey test, at a 5% probability.

## Results

### Variation in short- and long-term light intensity did not change net A of macaw palm seedlings

**Figure 1:**
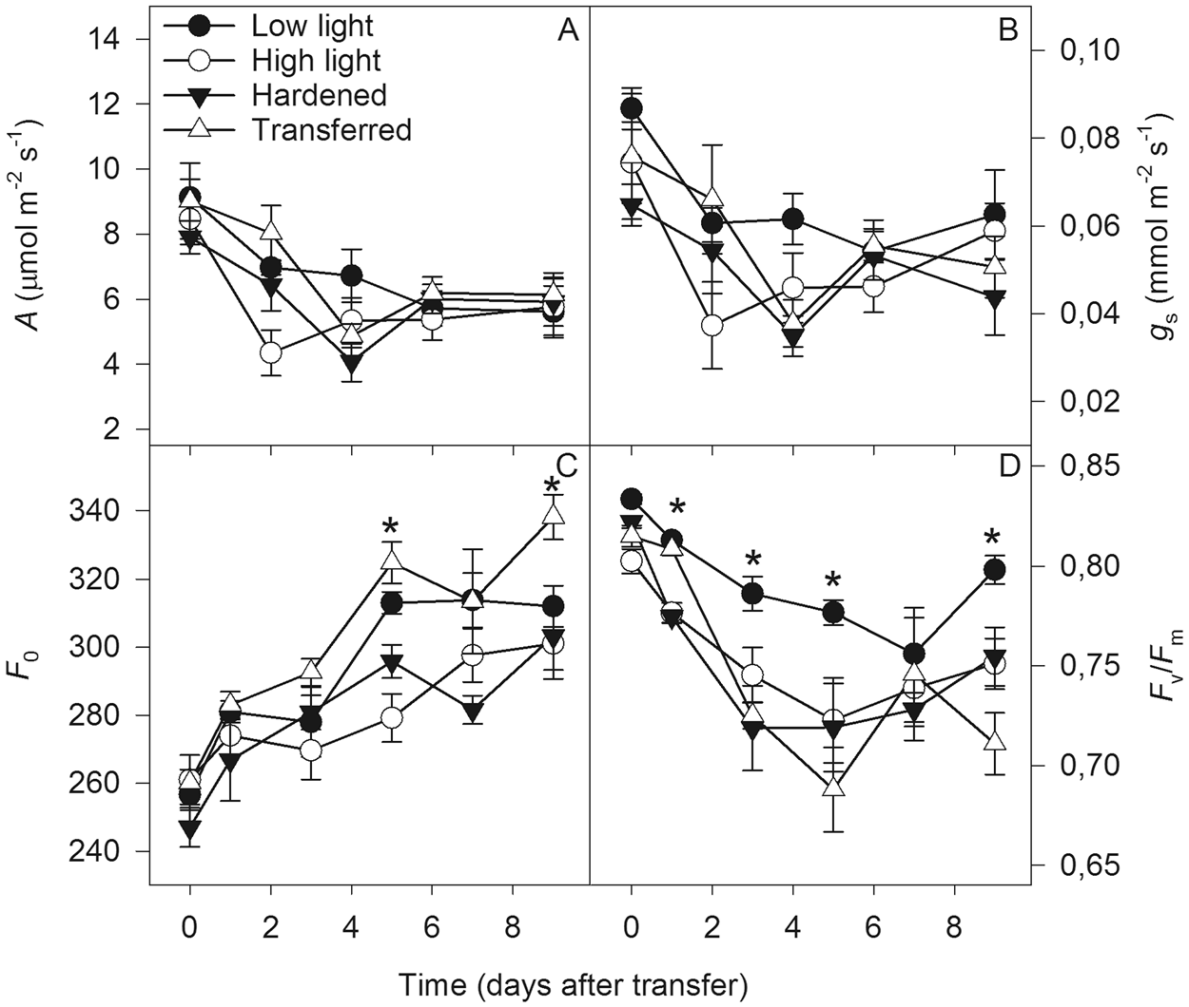
Photosynthesis (*A* – **A**) stomatal conductance (*g*_s_ – **B**) initial fluorescence (*F*_0_ – **C**) and maximum quantum efficiency of photosystem II (PSII; *F*_v_/*F*_m_ – **D**) in macaw palm seedlings grown in the short term under pre-nursery stage of contrasting light conditions. The bars indicate the standard error of the mean of five replicates and the asterisk indicates significant difference by the Tukey test, at 5% probability.

No significant changes were observed in the *A* and *g*_s_ (Fig. 1A,B) or *E*, *Ci*/*Ca* and *W*_t_ (Supplementary Fig. S1) of the macaw palm seedlings in response to the different lighting conditions or the sudden variation in light intensity under pre-nursery conditions. However, plants transferred directly from low to high light showed increased F_0_ with significant differences (p < 0.05) on the day 5 of evaluation when compared with the hardened and high light plants, and on day 9 with plants from the other treatments (Fig. 1C). In general, the low light seedlings showed significantly (p < 0.05) higher *F*_v_/*F*_m_ values compared to the other treatments (Fig. 1D). During the evaluation period, was observed a decrease in *A*, *g*_*s*_ and *F*_v_/*F*_m_, as a consequence of increasing *F*_0_, independent of the applied treatments (Fig. 1).

The seedlings grown under high light conditions showed significantly (p < 0.05) lower chlorophyll content indexes than seedlings that were transferred after 2 and 4 days did. No differences were found over time or among the other treatments (Supplementary Fig. S2). After 265-day long-term exposure to low and high light treatments, macaw palm plants showed significant (p < 0.05) difference in the levels of chlorophylls (*a*, *b*, and total) and carotenoid, with higher values found in low light plants. No significant changes were detected in the ratios of chlorophyll *a*/*b* and carotenoids/chlorophylls between treatments (Supplementary Fig. S3).

### Macaw palm presented high photochemical efficiency at low irradiance and effective energy dissipation under excessive light conditions

**Figure 2:**
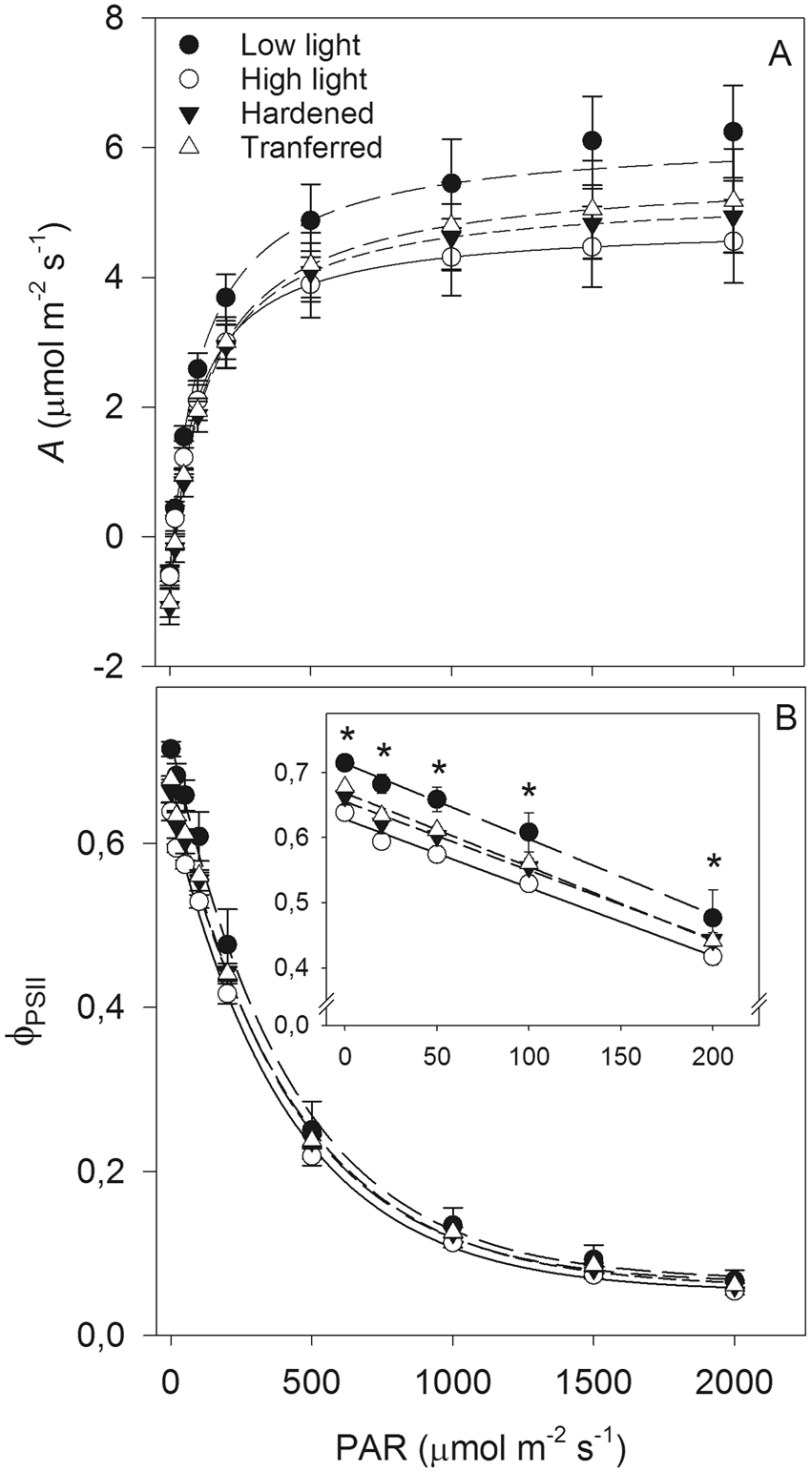
Light-response curves of net photosynthetic rate (*A* – **A**) and effective quantum yield of photosystem II (PSII; ϕ_PSII_ – **B**) in macaw palm seedlings grown in the short term under pre-nursery stage of contrasting light conditions. The inner panel indicates the magnified area from 0 to 200 µmol photons m^−2^ s^−1^. The bars indicate the standard error of the mean of five replicates and the asterisk indicates significant difference by the Tukey test, at 5% probability.

Plants from all the contrasting light conditions administered in the pre-nursery stage showed similar patterns in light response curves of the net *A* (Fig. 2A). Young macaw palm seedlings exhibited a mean *I*_max_ of 105 μmol m^−2^ s^−1^, high quantum efficiency in carbon fixation, and maximum net *A* of 5 μmol m^−2^ s^−1^ on average. No significant differences (p > 0.05) between treatments were observed for these variables (Table 1). However, plants that were directly transferred to high light and those subjected to the hardening procedure had a significantly higher dark respiratory rate (*R*_D_) than plants from other treatments did (Table 1). The plants maintained in the lower irradiance presented the lowest *I*_comp_ values, differing significantly from those of the hardened plants, which showed the highest values (Table 1).

**Table 1:** Light response curve variables from macaw palm seedlings grown in the short term under pre-nursery stage of contrasting light conditions.

Treatments	*R*_D_	*I*_comp_	*A*_max_	*I*_max_	ϕ_(*I*comp − *I*200)_
Shaded	0.6 ± 0.1 b	9.6 ± 1.5 b	5.8 ± 0.8 a	113.2 ± 18.8 a	0.017 ± 0.002 a
Full Sunlight	0.6 ± 0.1 b	13.4 ± 3.7 ba	4.5 ± 0.6 a	88.8 ± 12.7 a	0.014 ± 0.002 a
Hardened	1.1 ± 0.3 a	23.8 ± 5.9 a	4.9 ± 0.5 a	107.4 ± 11.1 a	0.015 ± 0.001 a
Transferred	1.0 ± 0.2 a	20.3 ± 1.7 ba	5.2 ± 0.8 a	110.2 ± 12.2 a	0.015 ± 0.001 a

*R*_D_ – dark respiration [μmol (CO_2_) m^−2^ s^−1^]; *I*_comp_ – light compensation point [μmol(photon) m^−2^ s^−1^]; *A*_max_ – maximum photosynthetic rate [μmol m^−2^ s^−1^]; *I*_max_ – light saturation point beyond which there is no significant change in net photosynthesis [μmol(photon) m^−2^ s^−1^]; ϕ_(*I*comp − *I*200)_ – maximum quantum yield at the range between *I*_comp_ and *I* = 200 μmol(photon) m^−2^ s^−1^ [μmol(CO_2_) μmol(photon)^−1^].

Means ± standard error followed by the same letter in a column do not differ from each other according to Tukey’s test at 5% significance level. (n = 5).

At low irradiance (<500 µmol m^−2^ s^−1^) the differences in the light response curve of the ϕ_PSII_ between the treatments were highlighted, with higher ϕ_PSII_ values shown by low light seedlings than by those exposed to high light treatment (Fig. 2B).

**Figure 3:**
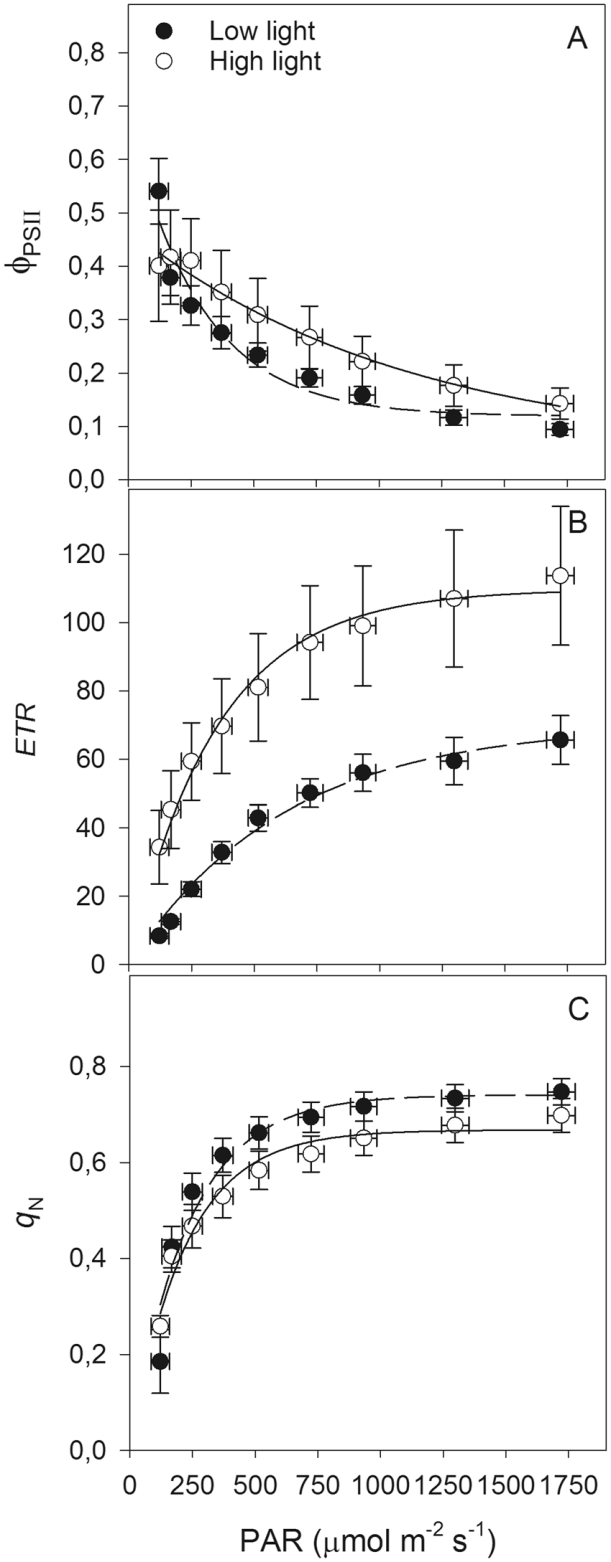
Light responses curves of the chlorophyll fluorescence variables: effective quantum yield of photosystem II (PSII; ϕ_PSII_ – **A**), relative electron transfer rate (*ETR* (µmol m^−2^ s^−1^) – (**B**) and non-photochemical quenching (*q*_N_ – **C**) in macaw palm seedlings grown in the long term under nursery conditions of low and high light. The bars indicate the standard error of the mean of five replicates. Significant differences (p < 0.05) were found in simple effects of treatments on the *ETR* and *q*_N_ variables.

In addition to the non-significant ϕ_PSII_ values between treatments (Fig. 3A), the long-term exposure (265 days) of seedlings to low and high light conditions changed the light response curve of some chlorophyll *a* fluorescence variables. Seedlings exposed to high light presented significantly (p < 0.05) higher *ETR* values than low light seedlings did (Fig. 3B), irrespective of irradiance level. In contrast, low light seedlings showed significantly higher (p < 0.05) energy dissipation through *q*_N_ mechanisms (Fig. 3C) and related to the higher ϕ_NPQ_ (see Supplementary Fig. S4). The *q*_L_ and ϕ_NO_ did not change significantly in response to the treatments (Supplementary Fig. S4).

### Effective photoinhibition recovery and photoprotection through dissipation of excess energy occurred in macaw palm seedlings

**Figure 4:**
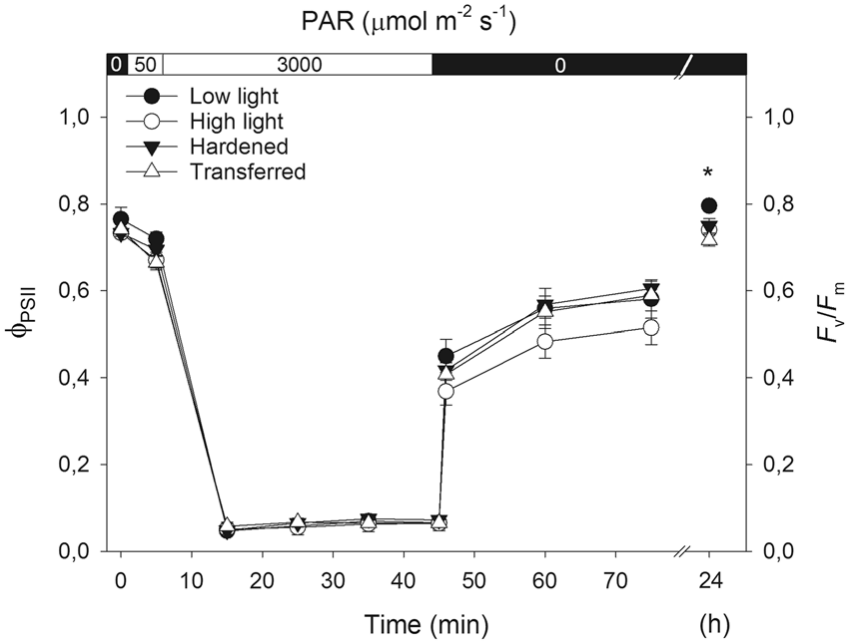
Photoinhibitory response of quantum yield of photosystem II in macaw palm seedlings grown short-term under pre-nursery stage of contrasting light conditions. The leaves were exposed to the following protocol: 5 min low light (50 µmol m^−2^ s^−1^), followed by 40 min high light (3000 µmol m^−2^ s^−1^), 30 min and 24 h of darkness. The bars indicate the standard error of the mean of five replicates and the asterisk indicates significant difference by the Tukey test, at 5% probability.

The macaw palm seedlings cultivated under contrasting light environments showed similar responses to the photosynthetic quantum yield during the initial exposure (5 min) to low light intensity (50 μmol m^−2^ s^−1^) and throughout the photoinhibitory process (40 min exposure to 3000 μmol m^−2^ s^−1^). During the dark-recovery period (46–75 min), values of the ϕ_PSII_ remained below the initial measurement of this variable (time zero), and the maximum recovery rate was achieved just after 1 min dark exposure. After the 24-h recovery period, the maximum photochemical efficiency values were re-attained, regardless of the treatments. Moreover, the seedlings from the low light treatment presented significantly higher (p < 0.05) *F*_v_/*F*_*m*_ values than seedlings from the other treatments did (Fig. 4).

**Figure 5:**
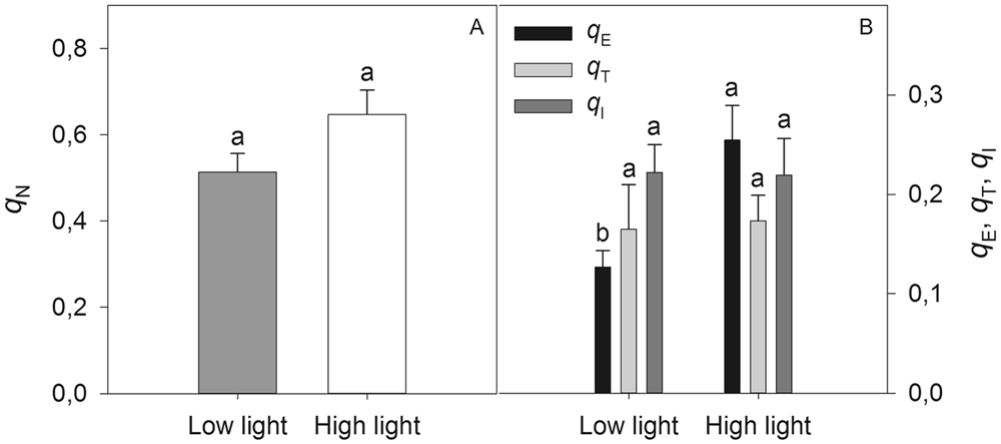
Non-photochemical quenching (*q*_N_ – **A**) and its three components (**B**) energy-dependent quenching (*q*_E_); state transition quenching of PSII (*q*_T_) and photoinhibitory quenching (*q*_I_) in young macaw palm plants grown long-term under nursery conditions of low and high light. The bars indicate the standard error of the mean of five replicates. Different letters indicate significant difference by the Tukey test, at 5% probability.

The exposure of young macaw palm plants to long-term low and high light treatments did not significantly change (p > 0.05) *q*_N_ (Fig. 5A). However, plants grown under high light presented a higher proportion of *q*_E_ than low light plants did (Fig. 5B). The other coefficients of non-photochemical quenching (*q*_T_ and *q*_I_) did not change in response to the treatments (Fig. 5B).

### Macaw palm presents limited morphoanatomic adjustments in response to light availability

**Figure 6:**
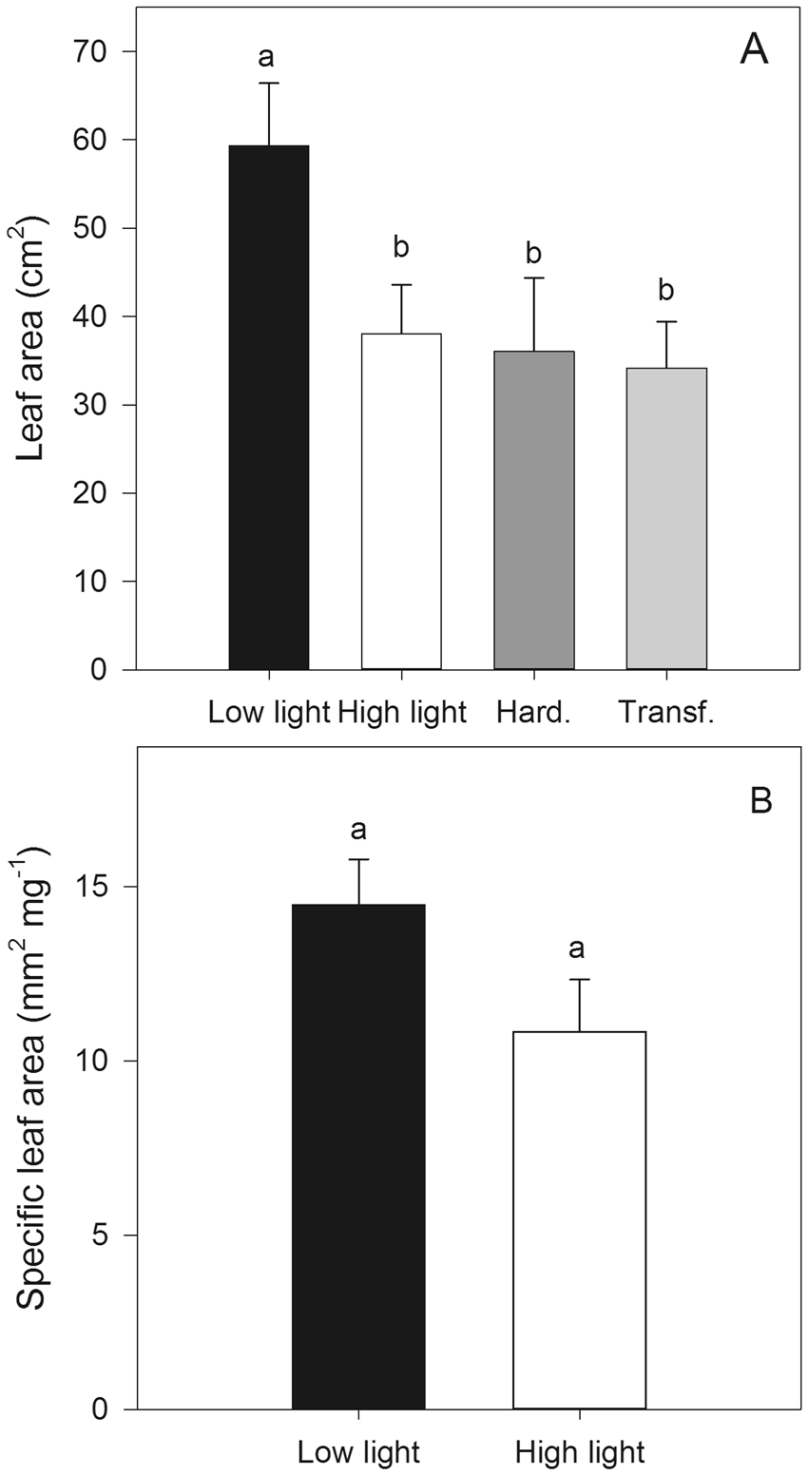
Leaf area (LA, **A**) of macaw palm seedlings grown in short-term under pre-nursery stage of contrasting light conditions and specific leaf area (SLA, **B**) of young macaw palm plants grown long-term under nursery conditions of low and high light. The bars indicate the standard error of the average of five replicates. Different letters indicate significant difference by the Tukey test, at 5% probability.

The macaw palm seedlings grown under low light conditions during the pre-nursery stage presented significantly larger (p < 0.05) LA than seedlings from the other treatments did, which did not differ among treatments (Fig. 6A). No significant differences (p > 0.05) were observed in the SLA after exposing young macaw palm plants to low and high light treatments for 265 days (Fig. 6B). However, periodical measurements of LA during the long-term exposure to different contrasting light conditions (Supplementary Fig. S5) showed a change in the LA pattern with shaded plants exhibiting higher values in the first 5 months than unshaded plants did. In addition, no significant differences between treatments were found during the remaining period (p > 0.05). Although the stipe increased during the development of the plantlets in the first year of life, the diameter and length of the stipe of the young macaw palm plants showed no significant difference between treatments until the end of the experiment when a greater stipe length was found in shaded plants than in those exposed to sunlight (Supplementary Fig. S5).

**Figure 7:**
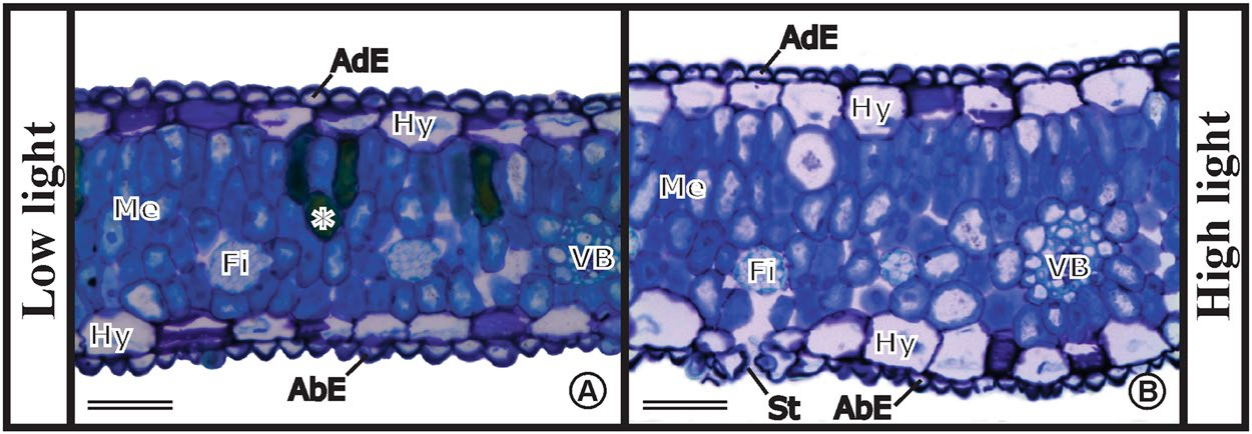
Cross sections of the leaflets from young macaw palm plants grown long-term under nursery conditions of low (**A**) and high light (**B**). AdE, adaxial surface epidermis; AbE, abaxial surface epidermis; Hy, hypodermis; Me, mesophyll; Fi, fiber; VB, vascular bundle; St, stoma; and (*) phenolic compounds. Scale bars = 50 µm (**A** and **B**).

Macaw palms display amphi-hypostomatic leaflets (stomata are present on both sides but with predominance in the abaxial surface), uniseriate epidermis and hypodermis on both sides, compact parenchyma with more elongated and narrow cells in the adaxial surface forming the palisade mesophyll, and they contain phenolic compounds (Fig. 7A and B). The vascular bundles are collateral and covered by layers of fibers and bundle sheath. After long-term exposure (265 days) to different light conditions, no significant changes (p > 0.05) in leaf tissue thickness of young macaw palm plants were observed (Table 2).

**Table 2:** Micromorphometric analysis of leaflets of young macaw palm plants grown in the long term under nursery conditions of low and high light.

Treatments	Thickness of leaf tissues (μm)					
	Adaxial surface		Abaxial surface		Mesophyll	Leaf blade
	Epidermis	Hypodermis	Epidermis	Hypodermis		
Low light	39.52 ± 0.94 a	63.18 ± 3.88 a	44.45 ± 2.63 a	61.93 ± 6.21 a	326.42 ± 24.26 a	523.60 ± 35.84 a
High light	37.73 ± 1.33 a	57.09 ± 3.55 a	40.50 ± 1.00 a	57.87 ± 6.07 a	309.53 ± 9.92 a	497.95 ± 12.15 a

Means ± standard error followed by the same letter in a column do not differ from each other according to Tukey’s test at 5% significance level. (n = 5).

## Discussion

The ability of macaw palm seedlings to maximize photosynthetic responses under contrasting light conditions is due to their specialized anatomical and physiological leaf features, consistent with pioneer species^7^. Irrespective of the incident light level during the initial growth period, macaw palm seedlings exhibit efficient mechanisms for maximizing light capture when lighting is low and dissipating excess energy to avoid damaging the PSII reaction center under supra-optimum irradiance conditions. Mainly due to energy dissipation trough non-photochemical quenching, macaw palm seedlings were able to maintain unaffected its net photosynthetic rate with high values of water use efficiency, regardless of the light treatment they were subjected to. These responses, optimize productivity and guarantee the establishment of the species in different environments. For photosynthetic organisms, it is essential to use the highest possible number of photons for the production of CO_2_, as well as to be able to quenching the excess energy that were not used, avoiding the transfer of this energy to reactive oxygen species, which are potentially toxic to cells^38^. *A*. *aculeata* is of natural occurrence in the Cerrado bioma, where the climate is predominantly savanna-like and the vegetation arbustive and grassy. Despite the fact that gas exchange measurements can be challenging in tropical dry regions, due to intrinsic variation of climatic variables, the palm is well-adapted; hence this rustic species probably possesses tolerance mechanisms to secure effective CO_2_ assimilation during drought or adverse climatic conditions, trough effective stomata control^19^.

Macaw palm seedlings presented some adjustments in the efficiency of light capture highlighted by the photosynthetic light response curves. The low *I*_max_ (105 μmol m^−2^ s^−1^) allows reaching the maximum potential for carbon fixation in relatively low light intensities, but without photosynthetic decay due to photoinhibition when exposed to high light intensity (up to 2000 μmol m^−2^ s^−1^). This photoinhibitory response to excess light would be very common in some palm species originally from dense forests phytophisionomies^9,39,40^. In addition to carbon gain, the maintenance of photosynthetic efficiency in response to contrasting light intensities would also be expressed as respiratory costs for maintaining a light compensation point in accordance with the availability of light in the environment^6^. Indeed, macaw palm seedlings grown under the shade minimized respiratory costs while keeping a positive carbon balance at low irradiance (>9.6 μmol m^−2^ s^−1^). Interestingly, the high-light-exposed seedlings also showed lower *R*_D_ than those that were transferred to full sunlight or hardened did, which indicates that the maintenance cost of the photosynthetic capacity of the former treatment would be lower. In fact, in this study, the hardening procedure did not appear to be advantageous during the pre-nursery stage of macaw palm seedling production since it did not promote better acclimation responses to contrasting light environments than the other methods did.

The efficiency of light capture under low light availability and the photosynthetic response of macaw palms to high light intensities was also confirmed by the results of Pires, *et al*.^18^ in the light response curves of field-grown plants during the reproductive stage. Sunlight-exposed field-grown macaw palm plants showed lower compensation irradiance and higher saturation irradiance^18^ than young macaw-palm seedlings did. In addition, seedlings of macaw palm plants in this study presented lower ϕ values for CO_2_ fixation (0.015 mol mol^−1^) than adult plants did, as reported previously^18^. The ϕ is a conservative variable, considering the light environment and the intrinsic traits of the leaves^41^ that did not exhibit significant changes among treatments. However, all these differences between seedlings and field-grown macaw palm plants indicate that the continuous adjustments of the light response curve variables are consistent with the variations in the light intensity and developmental stage^42^. While the ϕ remained unchanged in macaw palm seedlings cultivated under contrasting light environments, the higher ϕ_PSII_ of the shaded seedlings at low light intensities indicates that the light capturing and efficient use were linked to the carbon fixation at maximum photosynthetic efficiency. The values of ϕ_PSII_ obtained with rapid light-response curves were similar with those obtained by steady-state light curve at the same irradiance, which show the method’s reliability and allow better comprehension of the short-term acclimation changes in photosynthetic activity of macaw palm seedlings exposed to different light availability. At higher light intensities, the similar responses of ϕ_PSII_ for increasing PAR availability in both treatments, reveal that, regardless of its light growth environment, macaw palm seedlings capture and use quantum energy in the same capacity. On the other hand, the lower *ETR* response of low light plants indicating that as PAR availability increased, the plants grown and acclimated to low light setting could not effectively drawn the electrons trough photochemical processes. In such excess conditions, dissipation by heat is the ordinary mechanism to alleviate stress^38^ and, indeed, shaded macaw seedlings showed the higher *q*_N_ values at the expenses of electron transport trough PSII. Altogether, these phenomena reveal that the species modulated its physiology accordingly to the surroundings.

The higher *F*_v_/*F*_m_ observed in shaded seedlings than in those exposed to sunlight over the entire evaluation period, is a common feature in plants grown under low irradiance^12^, which maximizes light capture and promotes the efficient reduction of primary quinone electron acceptors in PSII^43^. The homogeneous response of *F*_v_/*F*_m_ among the other treatments without significant changes, indicates that the sudden exposure to high light intensity did not cause photodamage to the leaves of macaw palm seedlings. In contrast, the transference from shade to full sunlight conditions was accompanied by photooxidation and reactive oxygen species (ROS) overproduction in shade-tolerant dicot tree species from the Atlantic forest in Brazil^12^. In fact, no permanent photosynthetic damage occurred in macaw palm leaflets when transferred to high light, as corroborate the results on *C*_i_/*C*_a_ and chlorophyll content index, besides *F*_v_/*F*_m_ values. Chlorophyll molecules is very sensitive to photo-oxidative stress upon plant transference to excess light^6^, which may cause biochemical limitations of photosynthesis proven by increased *C*_i_/*C*_a_ values. However, this was not observed for macaw palm seedlings exposed to sudden high light intensity. The capacity for avoiding damage on PSII mainly in low light plants transferred to high irradiance might be due to its efficiency in dissipating excess energy through non-photochemical processes. In addition, without damage, the increase in *F*_0_ indicates a higher investment in a optimally organized and functional antenna system that are required for maximum *q*_N_ buildup^44^. The punctual increases in *F*_0_ observed in seedlings transferred to full sunlight after day 9 would indicate impairment in energy transfer from the antennae complex to the reaction center of the PSII. The decrease in *F*_v_/*F*_m_ and increase in *F*_0_ during the evaluation period would be the first symptoms of a leaf-age response^45^, although no significant reductions in chlorophyll were observed (Supplementary Fig. S1).

The acclimation of the macaw palm to different light conditions was evidenced by the chlorophyll contents in the leaflets. Different levels of luminosity during the initial growth of the seedlings results in high levels of photosynthetic pigments because there is a decrease in the available light intensity^10,46^. Nonetheless, the plants exposed to full sunlight maintained an efficient light capturing mechanism despite having lower chlorophyll contents than shaded plants did. The higher proportion of chlorophyll *b* in low light plants allows a greater uptake of light energy than in plants exposed to high light since the availability of this pigment influences the composition and size of the antenna complexes^47^. In addition to chlorophyll *b*, carotenoids are accessory pigments that play an important role in the photoprotection of PSII under excessive light conditions^48^. The high content of carotenoids in shaded macaw palm plants might have contributed to the effective non-photochemical energy dissipation. This mechanism involves the dissipation of excess energy that protects PSII reaction centers against light damage. Generally, leaves of plants grown in a shaded environment exhibited lower *q*_N_ than those grown under full sunlight conditions did^46,49^. However, for macaw palms, the plants grown in the low light environment showed the highest *q*_N_ values, especially under high light intensities during the light curve (Fig. 3), which protected the photosynthetic apparatus. Consequently, the plants avoided photoinhibition, which might have reduced the rate of accumulation of ROS such as singlet oxygen and superoxide radical anions^50^. This physiological adjustment contributed to the enhanced macaw palm growth under low irradiance condition, and the plants were able to establish themselves following exposure to high light intensity, such as conditions that occur with the presence of clearings in the forest canopy or cloudiness variation during the rainy season.

The excess energy dissipation in the photochemical apparatus depicted by *q*_N_ is the result of activation of various processes involved in the *q*_E_, *q*_T_ and *q*_I_^51^. When we evaluated the three components of *q*_N_ (*q*_E_, *q*_T_, and *q*_I_) in seedlings of macaw palm after long-term exposition to contrasting light intensities, the values of *q*_E_ under high light were greater than those under low light conditions, indicating that there was a greater dissipation of excess energy absorbed in the form of heat through the xanthophyll cycle. The xanthophylls are carotenoids involved in *q*_N_, and they act as antioxidants and protect the antenna complex from damage by excessive light^50^. While the *q*_T_ remained constant considering the high light exposure, the similar (non-significant) *q*_I_ in leaflets from plants in both treatments indicates that the photoprotective effect of the xanthophyll cycle was sufficient to prevent significant damage to the PSII even in shaded seedlings. It is noteworthy that the *q*_N_ partitioning in macaw palm was measured after exposure to excessive light levels that were above that necessary for photosynthetic saturation (3000 µmol m^−2^ s^−1^), which resulted in relatively high *q*_I_ values^43^.

The photochemical efficiency in contrasting light environments and the capacity to prevent photoinhibitory damage in macaw palm leaves was also demonstrated by the recovery of the properties after excess light treatment (Fig. 4). Moreover, activation of the fast mechanism for excess energy dissipation through *q*_E_ was efficient for a 65% recovery of the ϕ_PSII_ even after 1 min of high light exposure. However, 30 min in the dark was not sufficient to fully recover the maximum photochemical efficiency because of the photoinhibitory processes. Under excess irradiance photosynthesis may be severely inhibited, as observed with some vascular plant species^7,8,49^ including palms^9,40^. During this process, the highest levels of ROS are observed, and there may be permanent damage to the photosynthetic apparatus, known as chronic photoinhibition^50,52^. However, for the macaw palm, full recovery occurred 24 h after the excess light treatment, indicating the occurrence of dynamic photoinhibition, which is characterized by a temporary decrease in the maximum quantum efficiency of PSII^53^ and suggests a protective mechanism that deals with high levels of irradiance. Moreover, the maintenance of the photochemical integrity of the macaw palm under low light conditions was confirmed by the highest values of *F*_v_/*F*_m_ found after the full recovery period. The ability to overcome possible photoinhibitory damage and the magnitude of the response to a sudden increase in light intensity vary among plant species and are dependent on their ability to acclimate to this new condition^12^.

In addition to physiological adjustments, morphological changes also contribute to the acclimation responses to contrasting light conditions. The higher LA displayed during the initial growth of macaw palm seedling under the low light environment and its maximum photochemical efficiency, contribute to maximizing the light capture. However, during the transition from the pre-nursery stage to nursery-growing plants, with higher biomass accumulation^22^, the increase in the LA was similar between plants grown under contrasting light conditions, resulting in insignificant differences in the SLA. Generally, in dicot trees, shading increases the leaf height and LA compared to plants grown under full sunlight conditions^8,10^. As observed for young macaw palm plants, the light intensity did not affect the growth of other palm species, such as *Acrocomia emensis*, *Butia paraguayensis*, *and Syagrus petraea*^54^. The higher stipe length of the shaded macaw palm seedlings at the end of the assay may have been due to activation of some functional adjustments after long-term exposure to low light conditions^55^.

In contrast to the other plant species^10^, macaw palm seedlings did not exhibit significant anatomical changes in leaf tissues following exposure to contrasting light intensities. In response to increased light intensity, the differentiation of new layers or stretching of the cells of the palisade parenchyma and consequently increase in the thickness of the mesophyll are common^8,56^, as well as in the interception of light^11^. However, the greater elongation of mesophyll cells on the adaxial surface of macaw palm leaflets than on other surfaces appears to be a typical structural pattern of the species, regardless of the light condition.

Macaw palm seedlings exhibited limited leaflet anatomical plasticity, but they adjust to contrasting light conditions by modulating their light capturing efficiency under low light, and maximizing photosynthetic efficiency and non-photochemical energy dissipation under excess light conditions. The ability to recover rapidly from photoinhibition after sudden exposure to high irradiances and the long-term adjustments in chlorophyll content and leaf morphological traits allowed macaw palm establishment and cultivation under extremely different light conditions.

## Electronic supplementary material

Supplementary information
