# Supplementary material for: Acclimation responses of macaw palm seedlings to contrasting light environments
Source: Sci Rep. 2018 Oct 17;8:15300. doi: 10.1038/s41598-018-33553-1 (PMC6192989; doi:10.1038/s41598-018-33553-1)
Supplement: Supplementary file 1 — Supplementary information [file 41598_2018_33553_MOESM1_ESM.pdf]

Scientific Reports – Supplementary information

Acclimation responses of macaw palm seedlings to contrasting light environments

Adriel N. Dias[a], Advanio I. Siqueira-Silva[b,d], João P. Souza[b], Kacilda N. Kuki[c], and Eduardo G. Pereira[b*]

[a] Programa de Pós-graduação em Manejo e Conservação de Ecossistemas Naturais e Agrários, Universidade Federal de Viçosa (UFV), Campus Florestal - Florestal, Minas Gerais, Brazil

[b] Instituto de Ciências Biológicas e da Saúde, UFV, Campus Florestal - Florestal, Minas Gerais, Brazil

[c] Departamento de Fitotecnia, UFV, Campus Viçosa – Viçosa, Minas Gerais, Brazil

[d] Present address: Universidade Federal do Oeste do Pará (UFOPA), Campus Universitário de Juruti - Juruti, Pará, Brazil

* Corresponding author: UFV Campus Florestal, Postal Code: 35690-000, Florestal, Minas Gerais, Brazil; Tel.: +55 31 3536 3401; Fax: +55 31 3536 3361. E-mail address: egpereira@ufv.br (E.G. Pereira)

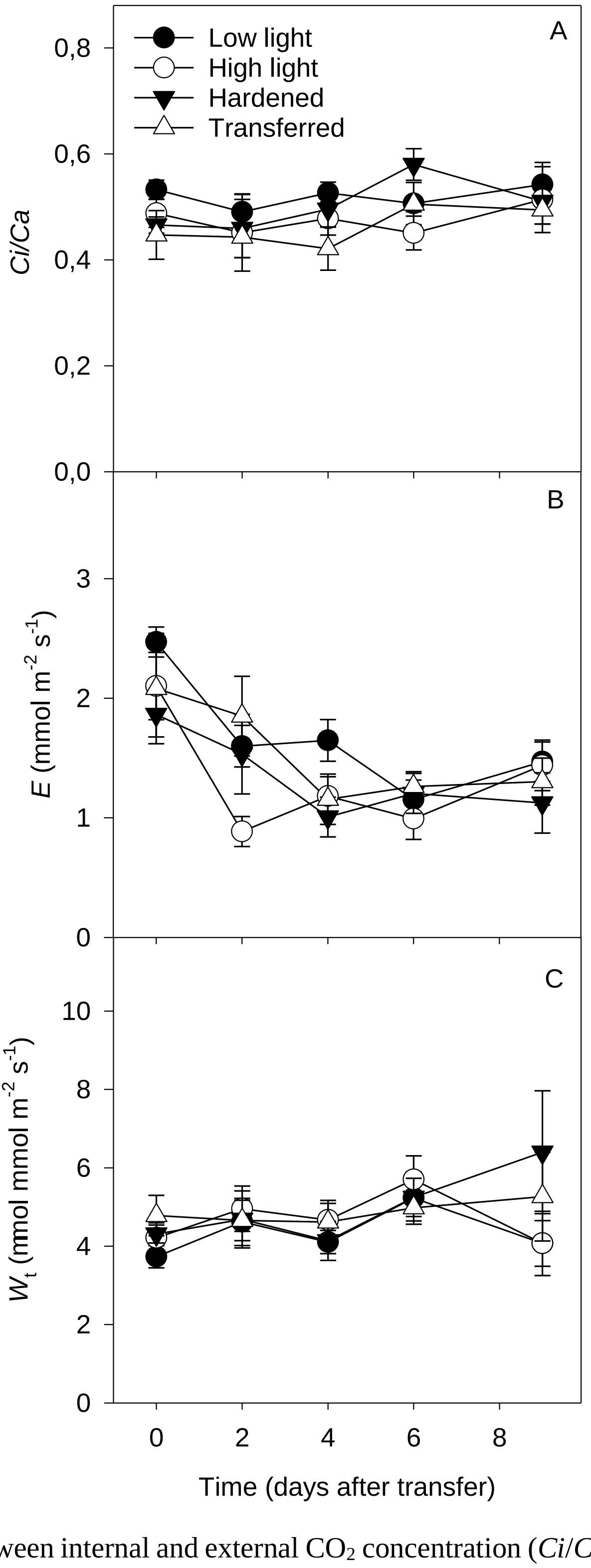

Fig. S1: The ratio between internal and external CO_2 concentration (*Ci*/*Ca* – A); transpiration rate (*E* – B); and instantaneous water use efficiency (W_t – C) in macaw palm seedlings grown in the short term under pre-nursery stage of contrasting light conditions. The bars indicate the standard error of the mean of five replicates. No significant differences (p > 0.05) were found between treatments.

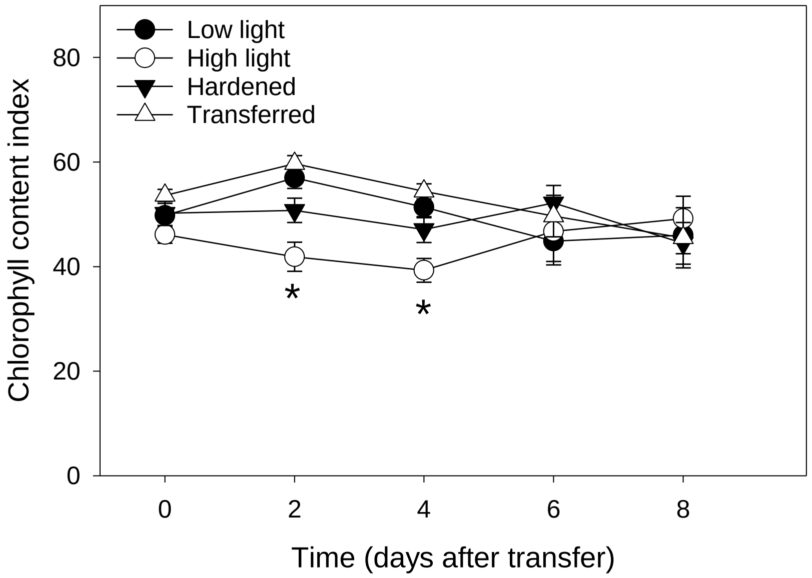

Fig. S2: Chlorophyll content index in macaw palm seedlings grown under short-term conditions in the pre-nursery stage of contrasting light conditions. The bars indicate the standard error of the mean of five replicates and the asterisk indicates significant difference by the Tukey test, at 5% probability.

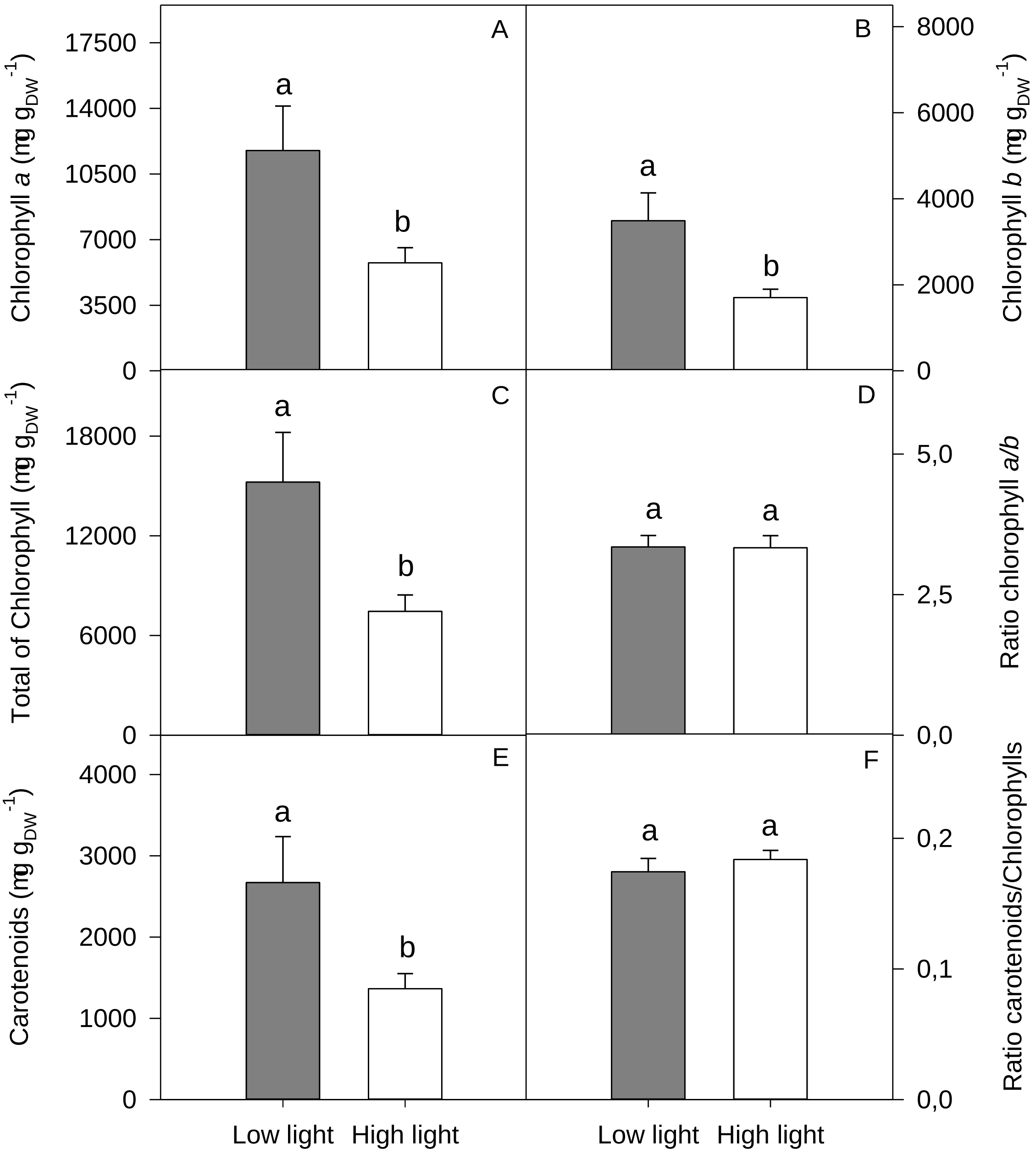

Fig. S3: Chlorophyll *a* (A), chlorophyll *b* (B), total of chlorophyll (C), ratio chlorophyll *a*/*b* (D), carotenoids (E) and ratio carotenoids/total chlorophylls (F) ratio in young macaw palm plants grown under long-term nursery conditions of low light and high light. The bars indicate the standard error of the mean of five replicates. Different lower-case letters indicates significant differences ($p < 0.05$) between treatments.

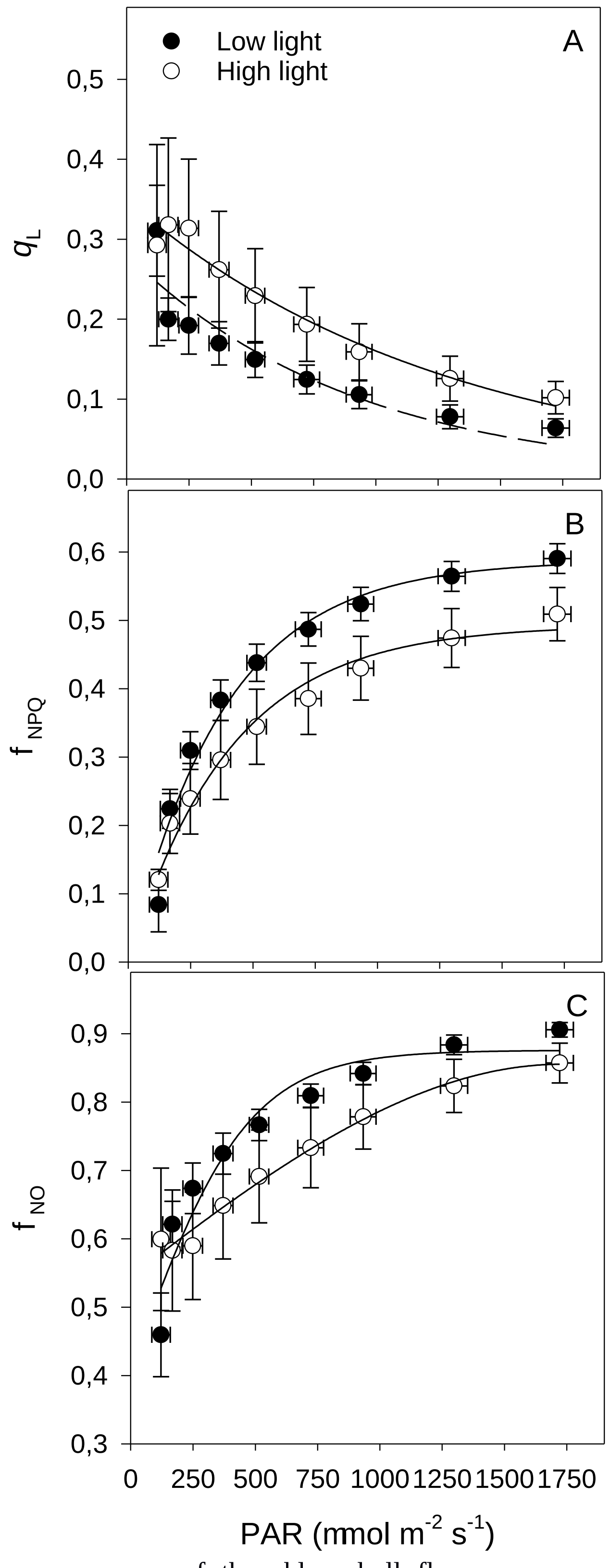

Fig. S4: Light responses curves of the chlorophyll fluorescence variables: photochemical quenching (q_L – A), quantum yield of regulated non-photochemical energy dissipation of PSII (ϕ_{NPQ} – B) and the quantum yield of non-regulated energy dissipation in PSII (ϕ_{NO} – C) in macaw palm seedlings grown long-term under nursery conditions of low light and high light. The bars indicate the standard error of the mean of five replicates. Significant differences ($p < 0.05$) were found to simple effects of treatments on the ϕ_{NPQ} variable.

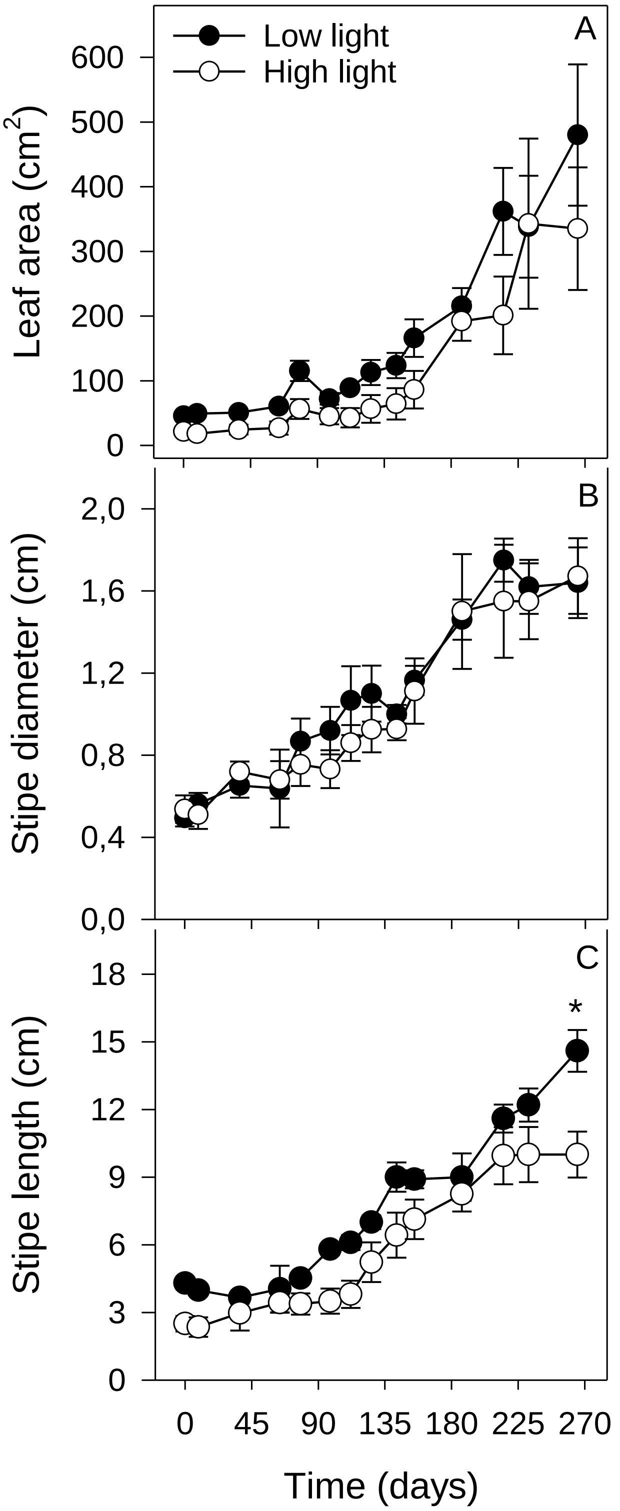

Fig. S5: Leaf area (LA, A); stipe diameter (B) and length (C) of young macaw palm plants grown under long-term nursery conditions of low light and high light. The bars indicate the standard error of the mean of five replicates and the asterisk indicates significant difference by the Tukey test, at 5% probability.
